# Cognitive Decline in Chronic Migraine with Nonsteroid Anti-inflammation Drug Overuse: A Cross-Sectional Study

**DOI:** 10.1155/2019/7307198

**Published:** 2019-05-06

**Authors:** Xiaoying Cai, Xiaotian Xu, Aiwu Zhang, Jianwen Lin, Xiaojuan Wang, Wen He, Yannan Fang

**Affiliations:** ^1^Department of Anesthesiology, The First Affiliated Hospital, Sun Yat-sen University, No. 58 Zhongshan 2nd Road, Guangzhou 510080, China; ^2^Guangdong Key Laboratory for Diagnosis and Treatment of Major Neurological Diseases, Department of Neurology, The First Affiliated Hospital, Sun Yat-sen University, No. 58 Zhongshan 2nd Road, Guangzhou 510080, China; ^3^Department of Neurology, Guangzhou First People's Hospital, No. 1 Panfu Road, Guangzhou 510080, China; ^4^Department of Geriatrics, The First Affiliated Hospital, Sun Yat-sen University, No. 58 Zhongshan 2nd Road, Guangzhou, Guangdong 510080, China

## Abstract

**Background:**

Chronic migraine with medication overuse headache (CM-MOH) is the most common type of chronic migraine, and it increases risk of stroke and white matter lesions. These pathologic changes could induce cognitive decline. However, the alteration of cognitive function in CM-MOH patients is not established. Therefore, we took this study to reveal the cognitive performances in CM-MOH.

**Methods:**

This cross-sectional study was conducted between December 2015 and January 2017. Patients were divided into CM-MOH, CMwoMOH (chronic migraine without medication overuse), and MO (migraine without aura) groups. Cognitive function was assessed in all cases during interictal periods using Addenbrooke's Cognitive Examination Test (ACE-R), Trail Making Test A/B (TMT A/B), and Digit Symbol Test (DST). Detailed headache characteristics and evaluation of anxiety, depression, and living and sleep quality were collected.

**Results:**

116 patients were included in this study. There were 21 CM-MOHs, 20 CMwoMOHs, 35 MOs, and 40 controls. Age and education were the independent risk factors of cognitive decline (*P* < 0.05). After adjusting, the risk of cognitive decline was higher in CM compared with control in ACE-R score and language fluency (*P* < 0.05). In addition, CM-MOH sufferers were in higher risk of memory and executive dysfunction (*P* < 0.05). The cognitive function had no difference between CM-MOH and CMwoMOH (*P* > 0.05). Meanwhile, CM-MOH got significantly higher scores than MO in anxiety and depression, with poorer performances in sleep and life quality (*P* < 0.05).

**Conclusion:**

The risk of cognitive decline increased in chronic migraine patients. Nonsteroid anti-inflammatory drugs overuse had no influence on cognitive performances among chronic migraine sufferers.

## 1. Introduction

Chronic migraine (CM) is a kind of repeated headache disorder, with a high disability rate among the population [1, 2]. This disorder is manifested as suffering of headache for more than 15 days per month, with no less than 8 days of migraine-like episodes. Also, these symptoms continue for at least 3 months [3]. Taking analgesic or triptans during acute attack of migraine is the most important factor for CM induction [4]. It is believed that chronic migraine with medication overuse headache (CM-MOH) is the most common type of this disease [5, 6]. It is usually accompanied by anxiety, depression, sleep disorder, and so on, leading to serious impact on living quality [7].

Repeated migraine episodes can increase the risk of cerebrovascular diseases, such as stroke and increasing white matter lesion [8–11]. These pathologies were known to be responsible for cognitive decline in migraineurs. So far, it has been demonstrated that these sufferers usually exhibit cognitive decline during acute attacks, including the deficit of executive function, language, visuospatial ability, and complex tasks [12, 13]. These might be attributed to the reduction of intracranial blood perfusion and the related changes during episodes. However, the cognitive performances recovered in the interictal periods, suggesting the reversibility of cognition among them [14]. In the chronification process, the alteration of cognitive performances is still not demonstrated.

CM-MOH is the most common form of chronic migraine and is usually accompanied with increased clinically silent lesions [15, 16]. Therefore, whether there was irreversible cognitive decline in CM-MOH patients during interictal periods remains to be revealed. This will help evaluate the cerebrovascular risk of CM-MOH patients, as well as provide guidance for clinical prevention and treatment.

## 2. Methods

### 2.1. Participants

This cross-sectional study was conducted between December 2015 and January 2017. Patients were recruited from the neurology outpatient clinic, the First Affiliated Hospital of Sun Yat-sen University. They were divided into three groups according to the criteria of the 3rd edition beta version of International Classification of Headache Disorders (ICHD-III beta) [17], including chronic migraine with medication overuse headache (CM-MOH), chronic migraine without medication overuse headache (CMwoMOH), and migraine without aura (MO) groups. A total of 116 cases participated in this study. There were 21 CM-MOHs, 20 CMwoMOHs, 35 MOs, and 40 controls. The included criteria were as follows: (a) diagnosis of episode migraine, CM with and without MOH based on the criteria of ICHD-III beta; (b) headache duration ≥ 1 year; (c) aged between 25 and 65; (d) confirmation of nonstructural lesions according to brain CT/MRI, in the interictal periods of migraine. The excluded criteria were as follows: (a) headache secondary to trauma, intracranial inflammation, brain tumor, and other neurological diseases; (b) existence of cerebrovascular disorders, neoplastic diseases, infectious diseases, rheumatic diseases, or connective tissue diseases; (c) unable to cooperate with the survey because of cognitive impairment or psychiatric disease. 40 controls were from the individuals who attended the hospital to consult for nonspecific complaints. Controls did not suffer from headaches or any other diseases and their neurologic examinations were normal. This study was approved by the Ethics Committee of the First Affiliated Hospital, Sun Yat-Sen University. All the subjects were required to sign informed consents prior to participation.

### 2.2. Basic Information

We collected demographical data of all participants at the first visit, including age, sex, education, job, weight, height, relevant medical history, and family history. A detailed questionnaire was used to record their headache characteristics, including headache years, location, headache nature, headache duration, comprised symptoms, headache frequency, monthly headache days, and monthly headache attacks. Analgesics used to control headache and their doses were recorded. For CM-MOH patients, monthly analgesic pills doses were calculated.

### 2.3. Neuropsychology Assessment

Several scales evaluating life quality and psychiatric status were conducted in patients. The Migraine Disability Assessment Test (MIDAS) was used to assess the headache frequency in three months and how often it limited their participation in daily activities. The Hamilton Anxiety Scale and Hamilton Depression Scale were used to display their mood state. The Short Form (36) Health Survey (SF-36) was used to evaluate the participants' health statement. Results of SF-36 were divided into eight sections, including vitality, physical functioning, bodily pain, general health perceptions, physical role functioning, emotional role functioning, social role functioning, and mental health. The Pittsburgh Sleep Quality Index (PSQI) was used to assess the sleep quality of participants.

### 2.4. Cognitive Evaluation

Addenbrooke's Cognitive Examination (ACE-R) is a set of tests for cognitive dysfunction screening. With a total score of 100 points, it is composed of five elements, including attention/orientation (18 points), memory (26 points), verbal fluency (14 points), language (26 points), and visuospatial abilities (16 points) [18]. It was wildly applied for the cognitive assessment of different diseases, such as Alzheimer's disease, Parkinson's disease, and vascular dementia. Meanwhile, it could detect mild cognitive dysfunction, with high sensitivity and specificity. Trail Making Test A + B (TMT A + B) and Digit Symbol Test (DST) are effective ways to examine the executive function. Therefore, we used a battery of screening test which included these three scales to clarify the cognitive function of CM-MOH sufferers.

To reduce the likelihood of fatigue in participants, after collecting basic information, we performed the cognitive evaluation at the sequence of ACE-R, TMT A + B, and DST, followed by neuropsychology assessment.

### 2.5. Statistical Analysis

All statistical analysis was performed on IBM SPSS 24.0 for Windows (SPSS Inc., Chicago, IL, USA). Categorical variables such as sex, education, smoking history, and alcohol history were presented with frequency. Continuous variables followed the normal distribution and were presented with mean ± standard deviation; otherwise, presented with median (interquartile range). One-way analysis of variance (ANOVA), Kruskal–Wallis test, and chi-squared tests were used to evaluate the differences among groups, respectively. For the pairwise comparison between groups, the Tukey–Kramer test after ANOVA and Mann–Whitney test after Kruskal–Wallis test were performed, following with Bonferroni correction of the *p* values. Binary logistic regression models were used to evaluate the risk factors of cognitive decline after filtering of the independent variable under univariate analysis. Odds ratios (ORs) and 95% confidence intervals (CIs) were calculated. A two-tailed *P* value < 0.05 was considered as statistically significant.

## 3. Result

### 3.1. Baseline Information

In this study, there were 116 participants, including 21 in CM-MOH, 20 in CMwoMOH, 35 in MO, and 40 in control. The baseline characteristics are shown in Table 1. There were no significant differences in age, sex, education, hypertension, diabetes, high low-density lipoproteinemia, smoke history, alcohol history, and family history among groups (*P* > 0.05).

All the cases in our study took NSAIDs, such as aminopyrine, phenacetin, aspirin, ibuprofen, and acetaminophen, to relieve headache. Most of them were compound preparation. The VAS score, drug dosages, and frequency were higher in CM-MOH, compared with CMwoMOH and MO, respectively (*P* < 0.05). The headache duration and family history had no significant difference among groups (*P* < 0.05) (Table 2).

### 3.2. Neuropsychology Assessment

As shown in Table 3, patients in CM-MOH got significantly higher scores than MO in anxiety and depression (*P* < 0.05). In addition, these scores in CMwoMOH had no statistical differences, compared with CM-MOH and MO groups, respectively (*P* > 0.05). MIDAS assessment had no statistical difference among groups (*P* > 0.05). SF-36 revealed that patients in CM-MOH and CMwoMOH got much lower scores than them in MO, with obvious differences (*P* < 0.05). PSQI showed that the sleep quality in CM-MOH was relatively worse than that in MO (*P* < 0.05).

### 3.3. Cognitive Function Assessment

The score of memory and TMT B were significantly reduced in CM-MOH, compared with MO and control (*P* < 0.05). However, the assessment of memory and TMT A + B did not reveal any significance in the comparison of CMwoMOH and CM-MOH, MO and control, respectively (*P* > 0.05). Scores of attention, language fluency, language, and visuospace had no statistical differences among groups (*P* > 0.05) (Table 4).

The low 20% performance of each cognitive score was defined as cognitive decline. Due to the narrow score width of MMSE and attention/orientation, they did not implement the partition of cognitive decline. The threshold of cognitive decline in other values was as follows: ACE-R ≤77 points, memory ≤20 points, language fluency ≤7 points, language ≤18 points, visuospace ≤14 points, TMT A ≥35.29 s, TMT B ≥95.86 s, and DST ≤25 points. In cognitive decline, the morbidity rate was higher in CM-MOH and CMwoMOH than in control, especially in ACE-R total score, language fluency, and executive function (*P* < 0.05). In addition, there was no statistical difference between MO and control in the morbidity rate of cognitive dysfunction (*P* > 0.05) (Table 5).

Univariate regression analysis revealed that age and education were the independent risk factors of cognitive decline (*P* < 0.05) (Supplementary table). Hypertension, high low-density lipoproteinemia, smoke history, alcohol history, anxiety, depression, MIDAS, and sleep quality were not the independent risk factors (*P* > 0.05). Therefore, three covariates, including different status of migraine, age, and education, were included in our final multivariate model. After age and education were adjusted, the risk of cognitive decline was higher in CM-MOH than in control for ACE-R score (OR = 8.52, 95% CI: 1.83–39.81, *P*=0.006), memory (OR = 6.92, 95% CI: 1.86–25.71, *P*=0.004), language fluency (OR = 7.67, 95% CI: 1.74–33.88, *P*=0.007), and executive function (TMT B OR = 50.80, 95% CI: 5.35–482.31, *P*=0.001). The risk of cognitive decline was higher in CMwoMOH than that in controls in ACE-R score (OR = 7.14, 95% CI: 1.50–34.04, *P*=0.014) and language fluency (OR = 8.24, 95% CI: 1.85–36.67, *P*=0.006). In order to evaluate the association between chronic migraine and cognitive dysfunction, we compared the cognitive function between CMwoMOH and MO. Results showed that there were not significant differences (*P* > 0.017). Meanwhile, we assessed the impact of analgesic overuse on the cognitive function of chronic migraine patients. In addition, our study found that the cognitive function had no differences between CM-MOH and CMwoMOH (*P* > 0.017) (Table 6).

## 4. Discussion

Our study indicated that CM patients had increased risk of cognitive decline, especially in language fluency. Besides, CM-MOH sufferers were in higher risk of memory and executive dysfunction. In addition, the cognitive function had no obvious differences between CMwoMOH and CM-MOH.

It has been illustrated that the cognitive function of migraine sufferers declined during acute attacks. This process may be due to the decreased regional blood flow during migraine episodes [19, 20] and the increased brain lesions, such as white matter lesions, and subclinical infarcts [11, 21]. Besides, the grey matter volume (GMV) of several brain areas, including prefrontal, cingulate cortex, right posterior parietal cortex, and orbitofrontal cortex, was decreased in migraine patients [22]. In addition, this change was associated with the increasing headache duration and frequency [23]. As these areas were related to pain conduction, it suggested that repeated acute attack of migraine could induce selective damage of the brain.

There were overlaps between pain conduction pathway and cognitive regions in the brain. For example, the anterior cingulate cortex could regulate selective attention, working memory, and ability of identifying mistakes [24]. The pain-related activation of insular cortex increased when the cognitive function declined. It indicated that the damage of the pain conduction pathway could result in changes of cognition. In addition, previous studies had found that there were structural lesions of pain conduction pathway in CM sufferers. The GMV of cingulate cortex, frontal cortex, and insular lobe was reduced in CM cases compared with episode migraine patients [25, 26]. Therefore, these structural abnormalities could be the reasons of cognitive changes in CM patients.

In our study, we found that the performance of language fluency was poor in CM. Our examination of language fluency consisted of phonemic and semantic elements [27]. In addition, the aim of the TMT B was to test the executive function-related attention, memory, processing speed, and thinking flexibility. The functional magnetic resonance image (fMRI) study has revealed that the neural circuit had some differences between phonemic and semantic fluency. The posterior segment of left inferior frontal gyrus was involved more in the circuit of phonemic fluency, while the anterior frontal lobe and posterior temporal lobe played a much more important role in the regulation of semantic fluency [28]. Moreover, CM-MOH intended to have an impairment of memory and executive function, which was mainly manifested in TMT B. Single-photon emission computerized tomography (SPECT) has found that the TMT B was associated with the function of the anterior cingulate cortex, corpus striatum, and thalamus [29]. These suggested that the dysfunction of language fluency and executive ability may be due to the pathological changes of relevant brain areas. The cognitive function decline caused by CM may be irreversible, and there were more lesions in the CM-MOH brain.

Long-term exposure in inflammation could impair cognitive function. The mechanism of it may be the direct damage from prostaglandin and the prostaglandin-induced suppression of amyloid-*β* clearage [30]. NSAIDs could break off this toxic effect through inhibition of the production of prostaglandin. However, the role of NSAIDs on cognition is still controversial. Some studies discovered that NSAIDs could improve cognition [31–33], while others found that NSAIDs had no effect [34–37]. Our previous study displayed that the white matter lesions was less and the level of inflammatory factor was lower in CM-MOH patients compared with CMwoMOH, indicating that the anti-inflammation role of NSAIDs could reduce white matter damage [15]. In the present study, we did not find any differences in cognitive performance between CM-MOH and CMwoMOH. This indicated that the brain lesion-related dysfunction of CM patients might be irreversible, and it exceeded the protective effect of NSAIDs. The cases in our study took NSAIDs as analgesic for headache; therefore, our results could not be applied for patients using triptans or ergotamine. It merited further analysis of the cognitive function when triptans or ergotamine was overused.

In addition, our study displayed that the estimation of anxiety and depression was severe in CM-MOH sufferers, as well as worse in life quality and sleep quality, compared with MO. Previous studies had found that constant suffering of anxiety, depression, or lack of sleep could influence cognition [38, 39]. Meanwhile, they could also induce migraine chronification [40]. Although, in our study, they were not the independent risk factors of cognitive decline under Univariate regression analysis, we should still pay attention to them in clinical practice.

Our study had some limitations. Firstly, more objective index of cognitive assessment, such as fMRI, was not obtained. Secondly, the sample size in our study was small. Last, as a cross-sectional design study, we could not evaluate the progress of cognitive decline in these patients. Further study with large sample size to assess the changes of cognitive performance with disease progress, especially after withdrawal of pain killers, is needed.

## 5. Conclusion

There was cognitive function decline in CM patients, both in CM-MOH and CMwoMOH. NSAIDs had no influence on the cognition of CM sufferers. Further studies are needed to trace the cognitive function in other types of CM-MOH.

## Figures and Tables

**Table 1:** Baseline characteristic of the participants.

		CM		MO	Control
		CM-MOH	CMwoMOH		
No.		21	20	35	40
Age, year, mean ± SD^a^		48.90 ± 13.51	48.40 ± 10.33	45.89 ± 7.10	47.10 ± 7.04
Female, %^b^		80.95	80.00	77.14	77.50
Education, year, %^b^	0–6	19.05	20.00	17.14	17.50
	7–9	52.38	50.00	51.43	52.50
	10–12	14.28	20.00	20.00	17.50
	>12	14.28	10.00	11.43	12.50
Hypertension, %^b^		14.28	10.00	0	0
Diabetes, %^b^		0	0	0	0
High LDL, %^b^		9.52	10.00	11.43	0
Smoke, %^b^		9.52	5.00	11.43	7.50
Alcohol, %^b^		0	0	2.86	2.50
Family history, %^b^		52.38	25.00	28.57	NA

^a^ANOVA, *P* > 0.05; ^b^chi-squared tests, *P* > 0.05. CM, chronic migraine; CM-MOH, chronic migraine with medication overuse headache; CMwoMOH, chronic migraine without medication overuse headache; MO, migraine without aura; SD, standard deviation; LDL, low-density lipoprotein; ANOVA, one-way analysis of variance.

**Table 2:** Headache characteristic of cases.

	CM		MO
	CM-MOH	CMwoMOH	
Headache years, mean ± SD^a^	22.67 ± 12.27	17.40 ± 10.68	12.54 ± 9.08
VAS, mean ± SD^b^	9.05 ± 1.24	7.30 ± 1.95	7.71 ± 1.51
Duration, day, mean ± SD^c^	1.05 ± 1.15	1.51 ± 0.78	1.63 ± 1.01
Headache frequency, days/month, median (IQR)^d^	30 (20–30)	20 (15–30)	3 (1–4)
Dosage, pills/attack, median (IQR)^e^	3 (2–6)	1 (0–1)	1 (0–1)
Analgesic frequency, days/month, median (IQR)^e^	30 (20–30)	1.5 (0–4.5)	1 (0–3)

^a^ANOVA, CM-MOH vs. MO, *P* < 0.05; CM-MOH vs. CMwoMOH, CMwoMOH vs. MO, *P* > 0.05. ^b^ANOVA, CM-MOH vs. CMwoMOH, CM-MOH vs. MO, *P* < 0.05; CMwoMOH vs. MO, *P* > 0.05. ^c^ANOVA, *P* > 0.05. ^d^Kruskal–Wallis tests, *P* < 0.001; pairwise comparison with adj. sig., CM-MOH vs. MO, CMwoMOH vs. MO, *P* < 0.001; CM-MOH vs. CMwoMOH, *P* > 0.05. ^e^Kruskal–Wallis tests, *P* < 0.001; pairwise comparison with adj. sig., CM-MOH vs. CMwoMOH, CM-MOH vs. MO, *P* < 0.001; CMwoMOH vs. MO, *P* > 0.05; CM, chronic migraine; CM-MOH, chronic migraine with medication overuse headache; CMwoMOH, chronic migraine without medication overuse headache; MO, migraine without aura; SD, standard deviation; IQR: interquartile range; ANOVA, one-way analysis of variance.

**Table 3:** Neuropsychological assessment of cases^a^.

		CM		MO
		CM-MOH	CMwoMOH
Anxiety^b,1,3^		12 (5–16.5)	6 (4.3–8)	4 (3–6)
Depression^b,1,2^		4 (2.5–13.5)	2 (2–3)	1 (0–2)
MIDAS		0 (0–180)	12 (0–47.3)	6 (3–18)
SF-36	Physical functioning^b^	90 (85–95)	90 (75–93.8)	95 (90–100)
	Physical role	75 (12.5–100)	50 (25–75)	50 (25–100)
	Body pain	40 (22–68)	51 (42–54)	51 (41–74)
	General health^b,1,3^	45 (20–50)	30 (20–48.8)	52 (40–70)
	Vitality^b,1,3^	55 (40–75)	50 (45–60)	80 (60–80)
	Social role^b^	67 (44–89)	72.5 (56–78)	78 (78–89)
	Emotional role^b,1^	33 (16.5–33)	49.5 (33–66)	100 (33–100)
	Mental health^b,3^	60 (48–76)	50 (48–61)	72 (56–80)

PSQI	Overall sleep quality	2 (1–2)	1 (1–1.8)	1 (1–2)
	Sleep latency	2 (1–3)	1 (0–2.8)	1 (0–2)
	Duration of sleep	1 (0–2)	1 (0–1)	0 (0–1)
	Sleep efficiency^b,1,2^	1 (0–1)	0 (0–0)	0 (0–0)
	Sleep disturbance	1 (1–2)	1 (1–1)	1 (1–1)
	Need meds to sleep	0 (0–1.5)	0 (0–0)	0 (0–0)
	Day dysfunction due to sleepiness	1 (0–1)	0 (0–1)	0 (0–1)
	Total^b,1^	7 (5–9.5)	4.5 (3–7)	5 (3–7)

^a^Kruskal–Wallis tests, median (interquartile range); ^b^ *P* < 0.05. Pairwise comparison with adj. sig.: ^1^ CM-MOH vs. MO, *P* < 0.05. ^2^CM-MOH vs. CMwoMOH, *P* < 0.05. ^3^CMwoMOH vs. MO, *P* < 0.05; CM, chronic migraine; CM-MOH, chronic migraine with medication overuse headache; CMwoMOH, chronic migraine without medication overuse headache; MO, migraine without aura; MIDAS, Migraine Disability Assessment Test; SF-36, Short Form (36) Health Survey; PSQI, Pittsburgh Sleep Quality Index.

**Table 4:** Scores of cognitive function assessment in different groups^a^.

	CM		MO	Control
	CM-MOH	CMwoMOH		
MMSE	29 (27–29.5)	28.5 (28–29)	28 (28–29.5)	29 (28–30)
ACE-R	83 (74.5–88)	83 (76.3–88.5)	86 (78–92)	86 (82.3–89.8)
Attention/orientation	18 (17–18)	17 (17–18)	17 (17–18)	18 (17–18)
Memory^b,1,2^	21 (18–23)	21.5 (19.3–23)	24 (21–25)	23 (22–24)
Language fluency	8 (7–9)	8 (7–9)	9 (7–11)	9 (8–10)
Language	23 (18.5–24.5)	20 (18–22)	20 (18–23)	21 (19–24)
Visuospace	15 (13.5–16)	15.5 (14.3–16)	16 (15–16)	16 (15–16)
TMT A	50.3 (41.2–76.7)	50.3 (35.1–75)	45.1 (34.9–59.4)	48.2 (39.7–59.3)
TMT B^b,1,2^	145.3 (119.2–198.9)	111.2 (87.9–151.7)	116.3 (97.5–129.4)	119.6 (98.5–126.5)
DST	31 (23.5–45.5)	32 (20–41.3)	37 (30–45)	35.5 (30–42.8)

^a^Kruskal–Wallis tests, median (interquartile range); ^b^ *P* < 0.05. Pairwise comparison with adj. sig.:^1^ CM-MOH vs. control, *P* < 0.05. ^2^ CM-MOH vs. MO, *P* < 0.05. ^3^CM-MOH vs. CMwoMOH, *P* < 0.05; CM, chronic migraine; CM-MOH, chronic migraine with medication overuse headache; CMwoMOH, chronic migraine without medication overuse headache; MO, migraine without aura; MMSE, minimental state examination; ACE-R, Addenbrooke's Cognitive Examination Test; TMT, Trail Making Test; DST, Digit Symbol Test.

**Table 5:** Morbidity of cognitive decline in different groups^a^.

	CM		MO	Control
	CM-MOH	CMwoMOH		
ACE-R^1^	8 (38.1)	7 (35.0)	6 (17.1)	3 (7.5)
Memory^1^	10 (47.6)	6 (30.0)	7 (20.0)	5 (12.5)
Language fluency^1^	8 (38.1)	8 (40.0)	9 (25.7)	3 (7.5)
Language	5 (23.8)	6 (30.0)	14 (40.0)	8 (20.0)
Visuospace	8 (38.1)	5 (25.0)	6 (17.1)	6 (15.0)
TMT A	8 (38.1)	5 (25.0)	6 (17.1)	4 (10.0)
TMT B^1^	11 (52.4)	5 (25.0)	6 (17.1)	1 (2.5)
DST^1^	7 (33.3)	8 (40.0)	3 (8.6)	5 (12.5)

The low 20% performance of each cognitive evaluation was defined as cognitive decline. ^a^Chi-squared tests, cases of cognitive decline (%). ^1^Chi-squared tests, *P* < 0.05; CM, chronic migraine; CM-MOH, chronic migraine with medication overuse headache; CMwoMOH, chronic migraine without medication overuse headache; MO, migraine without aura; ACE-R, Addenbrooke's Cognitive Examination Test; TMT, Trail Making Test; DST, Digit Symbol Test.

**Table 6:** Risk factor analysis of cognitive decline after adjustment.

	*N*	Cognitive decline	Adjusted OR (95% CI)	*P*
*ACE-R*				
Control (reference)	40	3	1.00	—
CM-MOH	21	8	8.52 (1.83–39.81)	**0.006**
CMwoMOH	20	7	7.14 (1.50–34.04)	**0.014**
MO	35	6	2.72 (0.60–12.28)	0.194
MO (reference)	35	6	1.00	—
CMwoMOH	20	7	2.63 (0.68–10.14)	0.161
CMwoMOH (reference)	20	7	1.00	—
CM-MOH	21	8	1.19 (0.31–4.67)	0.799

*Memory*				
Control (reference)	40	5	1.00	—
CM-MOH	21	10	6.92 (1.86–25.71)	**0.004**
CMwoMOH	20	6	3.05 (0.77–12.00)	0.112
MO	35	7	1.80 (0.50–6.41)	0.370
MO (reference)	35	7	1.00	—
CMwoMOH	20	6	1.70 (0.46–6.23)	0.427
CMwoMOH (reference)	20	6	1.00	—
CM-MOH	21	10	2.27 (0.60–8.57)	0.226

*Language fluency*				
Control (reference)	40	3	1.00	—
CM-MOH	21	8	7.67 (1.74–33.88)	**0.007**
CMwoMOH	20	8	8.24 (1.85–36.67)	**0.006**
MO	35	9	4.39 (1.07–17.96)	0.040
MO (reference)	35	9	1.00	—
CMwoMOH	20	8	1.88 (0.57–6.19)	0.301
CMwoMOH (reference)	20	8	1.00	—
CM-MOH	21	8	0.93 (0.26–3.33)	0.912

*Language*				
Control (reference)	40	8	1.00	—
CM-MOH	21	5	1.25 (0.34–4.64)	0.742
CMwoMOH	20	6	1.72 (0.48–6.20)	0.405
MO	35	14	2.91 (1.00–8.51)	0.051
MO (reference)	35	14	1.00	—
CMwoMOH	20	6	0.59 (0.17–2.03)	0.592
CMwoMOH (reference)	20	6	1.00	—
CM-MOH	21	5	0.72 (0.17–3.05)	0.660

*Visuospace*				
Control (reference)	40	6	1.00	—
CM-MOH	21	8	3.94 (1.04–14.99)	0.044
CMwoMOH	20	5	1.86 (0.45–7.75)	0.393
MO	35	6	1.21 (0.33–4.41)	0.776
MO (reference)	35	6	1.00	—
CMwoMOH	20	5	1.54 (0.37–6.49)	0.554
CMwoMOH (reference)	20	5	1.00	—
CM-MOH	21	8	2.12 (0.49–9.10)	0.314

*TMT A*				
Control (reference)	40	4	1.00	—
CM-MOH	21	8	3.25 (0.64–16.54)	0.156
CMwoMOH	20	5	1.63 (0.31–8.61)	0.564
MO	35	6	2.18 (0.52–9.11)	0.287
MO (reference)	35	6	1.00	—
CMwoMOH	20	5	0.99 (0.22–4.50)	0.987
CMwoMOH (reference)	20	5	1.00	—
CM-MOH	21	8	1.83 (0.36–9.22)	0.466

*TMT B*				
Control (reference)	40	1	1.00	—
CM-MOH	21	11	50.80 (5.35–482.31)	**0.001**
CMwoMOH	20	5	11.30 (1.14–111.95)	0.038
MO	35	6	8.82 (0.97–79.82)	0.053
MO (reference)	35	6	1.00	—
CMwoMOH	20	5	1.28 (0.30–5.51)	0.739
CMwoMOH (reference)	20	5	1.00	—
CM-MOH	21	11	4.50 (0.98–20.54)	0.052

*DST*				
Control (reference)	40	5	1.00	—
CM-MOH	21	7	2.83 (0.67–11.91)	0.157
CMwoMOH	20	8	4.25 (1.06–17.09)	0.041
MO	35	3	0.65 (0.14–3.04)	0.580
MO (reference)	35	3	1.00	—
CMwoMOH	20	8	6.59 (1.35–32.08)	0.020
CMwoMOH (reference)	20	8	1.00	—
CM-MOH	21	7	0.67 (0.16–2.84)	0.582

All the data were adjusted by age and education. The low 20% performance of each cognitive evaluation was defined as cognitive decline. *P* < 0.017 (0.05/3) was considered as significant difference; CM, chronic migraine; CM-MOH, chronic migraine with medication overuse headache; CMwoMOH, chronic migraine without medication overuse headache; MO, migraine without aura; *N*, number of cases; OR, odds ratio; CI, confidence interval; ACE-R, Addenbrooke's Cognitive Examination Test; TMT, Trail Making Test; DST, Digit Symbol Test.

## Data Availability

The datasets supporting the conclusions of this article are included within the article.

## Supplementary Materials

Supplementary Materials Supplementary table: risk factor analysis of cognitive decline (univariate regression analysis).
