# Supplementary material for: Cognitive Decline in Chronic Migraine with Nonsteroid Anti-inflammation Drug Overuse: A Cross-Sectional Study
Source: Pain Res Manag. 2019 May 6;2019:7307198. doi: 10.1155/2019/7307198 (PMC6526623; doi:10.1155/2019/7307198)
Supplement: Supplementary Materials — Supplementary table: risk factor analysis of cognitive decline (univariate regression analysis). [file 7307198.f1.pdf]

Supplementary Table: Risk factor analysis of cognitive decline (Univariate regression analysis)

		**Age**		**Education**	
		***OR* (95%*CI*)**	***P***	***OR* (95%*CI*)**	***P***
	ACE-R	1.02 (0.97-1.07)	0.388	0.41 (0.22-0.79)	**0.007**
	Memory	1.01 (0.96-1.05)	0.810	0.55 (0.32-0.96)	**0.036**
	Language	0.99 (0.95-1.04)	0.831	0.47 (0.27-0.82)	**0.008**
	Visuospace	1.02 (0.97-1.07)	0.518	0.33 (0.17-0.66)	**0.002**
Executive function	TMT A	1.15 (1.08-1.22)	**0.000**	0.83 (0.49-1.42)	0.504
	TMT B	1.07 (1.02-1.13)	**0.010**	0.50 (0.27-0.93)	**0.029**
	DST	1.09 (1.03-1.15)	**0.002**	0.55 (0.31-1.01)	0.052

The low 20% performance of each cognitive evaluation was defined as cognitive decline.

Abbreviation: CM, chronic migraine; CM-MOH, chronic migraine with medication overuse headache; CMwoMOH, chronic migraine without medication overuse headache; MO, migraine without aura; OR, odds ratio; CI, confidence interval; ACE-R, Addenbrooke's cognitive examination test; TMT, Trail Making Test; DST, Digit Symbol Test.
